# Postoperative Pulmonary Complications After Laparoscopic Surgery: Risk Factors and Predictive Scores

**DOI:** 10.3390/medicina61091635

**Published:** 2025-09-10

**Authors:** Lucía Valencia, Ángel Becerra-Bolaños, Rocío Rodríguez-Sánchez, Nazario Ojeda, Aurelio Rodríguez-Pérez

**Affiliations:** 1 Department of Anaesthesiology and Intensive Care, Hospital Universitario de Gran Canaria Doctor Negrín, 35010 Las Palmas de Gran Canaria, Spainnojebet@gobiernodecanarias.org (N.O.); arodperp@gobiernodecanarias.org (A.R.-P.); 2 Department of Medicine, University of Fernando Pessoa Canarias, 35010 Las Palmas de Gran Canaria, Spain; 3 Department of Medical and Surgical Sciences, Universidad de Las Palmas de Gran Canaria, 35010 Las Palmas de Gran Canaria, Spain; 4 Department of Medicine, Complejo Hospitalario Universitario Insular-Materno Infantil, 35010 Las Palmas de Gran Canaria, Spain

**Keywords:** postoperative complication, laparoscopic surgery, general anesthesia

## Abstract

*Background*: Laparoscopic surgery is associated with postoperative pulmonary complications (PPCs) that may lead to increased morbidity and prolonged hospital stay. This study aimed to identify risk factors for PPCs within the first 7 days following laparoscopic surgery. *Methods*: We conducted a prospective observational study including patients scheduled for laparoscopic surgery between June 2021 and June 2024. The primary endpoint was the incidence of PPCs, defined according to the Joint Task Force of the European Society of Anaesthesiology and the European Society of Intensive Care Medicine (ESA and ESICM). Secondary endpoints included other postoperative complications as well as hospital and post-anesthesia care unit (PACU) length of stay. Demographic data, intraoperative variables, Air Test, incidence of PPCs, and hospital length of stay were collected. Logistic regression analysis was performed to identify factors associated with the development of PPCs. *Results*: A total of 250 patients were included in the study. PPCs occurred in 34 patients (14.4%). Laparoscopic upper abdominal surgeries (*p* = 0.086) and longer surgical duration (*p* = 0.025) were associated with a higher incidence of PPCs. Independent risk factors identified for PPCs were age over 60 years (OR: 2.29; 95% CI 1.03–5.08; *p* = 0.041) and a positive Air Test result (OR: 6.22; 95% CI 2.11–18.22; *p* = 0.001). Patients in the PPC group had significantly higher rates of postoperative infectious complications, as well as longer stays in both the post-anesthesia care unit (PACU) and the hospital. The Air Test demonstrated acceptable discriminative performance, with an area under the ROC curve (AUC) of 0.66 (95% CI: 0.58–0.74; *p* = 0.002). *Conclusions*: The incidence of PPCs in patients undergoing laparoscopic surgery was 14.4%. Factors independently associated with PPCs included advanced age and a positive postoperative Air Test. However, the Air Test demonstrated modest accuracy in predicting PPCs.

## 1. Introduction

Laparoscopic surgery has advanced significantly in recent years and is now the preferred approach for most abdominal procedures. This technique offers several advantages over open surgery, including reduced postoperative pain, and faster recovery times [1]. It also offers specific benefits in laparoscopic surgery for endometrioma excision, particularly regarding the impact of the hemostatic approach on ovarian reserve. [2]. One of the main improvements of laparoscopy over laparotomy is the reduction in postoperative pulmonary complications (PPCs). This benefit has been demonstrated in various settings, including hepatectomy [3] and bariatric laparoscopic surgery [4]. In patients at high risk for PPCs (such as those with obesity [4] or chronic obstructive pulmonary disease [5]), laparoscopic surgery significantly reduces the incidence of these complications compared to open surgical approaches.

However, despite its overall favorable profile regarding PPCs, laparoscopic surgery is not without risk [6]. The insufflation of intraperitoneal carbon dioxide (CO_2_) at high pressures, combined with the Trendelenburg position, can contribute to the development of PPCs, particularly atelectasis and pneumonia, two of the most common respiratory issues following laparoscopic procedures [7].

It is important to emphasize that even mild respiratory complications are associated with increased early postoperative mortality, increased intensive care unit (ICU) admission, and prolonged hospital stay. PPCs are reported in up to 40% of patients undergoing elective abdominal surgery [8]. In the setting of emergency laparotomy, the incidence remains notably high, approximating 22% [9]. Data regarding the incidence of PPCs in laparoscopic general surgery are limited [10,11,12].

Risk stratification tools have been developed to identify patients at increased risk of PPCs. The ARISCAT score (Assess Respiratory Risk in Surgical Patients in Catalonia) is one of the most widely used tools currently available [13]. The ARISCAT score uses seven variables, each assigned a point value. The total score stratifies patients into low, intermediate, or high risk for PPCs (1.6, 13, and 46%, respectively). Another recently developed tool is the Air Test, which evaluates arterial oxygen saturation (SpO_2_) by pulse oximetry after 5 min of breathing room air (FiO_2_ 21%) performed 30 min after extubation following abdominal surgery [14]. A positive Air Test, defined as SpO_2_ ≤ 96%, has been shown to be indicative of atelectasis. However, there is limited literature on the usefulness of predictive factors for PPCs and specific risk scores in patients undergoing laparoscopic surgery. Due to its minimally invasive nature compared to laparotomy, laparoscopic surgery represents a distinct clinical context that justifies separate evaluation regarding the risk of PPCs.

The primary objective of this study was to determine the incidence of PPCs during the first seven postoperative days and to identify independent risk factors associated with their development in patients undergoing laparoscopic surgery. Postoperative outcomes, including lengths of stay in the hospital and in the post-anesthesia care unit (PACU), were also compared between patients who developed PPCs (PPC group) and those who did not (non-PPC group).

## 2. Materials and Methods

After obtaining approval from the Ethics Committee of the Hospital Universitario de Gran Canaria Doctor Negrín (approval #2019-484-1, on 2 December 2019) and prospectively registering in ClinicalTrials.gov (NCT04895527), we conducted a prospective observational study. Inclusion criteria were patients aged 18 years and older, scheduled for laparoscopic surgery between June 2020 and June 2024. Written informed consent was obtained from each patient. Exclusion criteria were conversion to laparotomy, pregnancy, uncooperative or agitated patients in the immediate postoperative period, history of lung resection, and refusal to participate in the study.

### 2.1. Routine Peri-Procedural Management

Upon arrival at the operating room, demographic data (age, gender, ideal body weight) and American Society of Anesthesiologists (ASA) physical status were recorded. Body mass index (BMI), ARISCAT risk index, and FRAIL scale were calculated. The FRAIL scale is used to screen for frailty, particularly in older adults, and helps predict adverse outcomes such as postoperative complications, prolonged recovery, and increased mortality. The scale predicted body weight was calculated in each patient as follows: females: 45.5 + 0.91 × (height in cm − 152); males: 50 + 0.91 × (height in cm − 152). Comorbidities were also recorded. Indications for laparoscopic surgery and baseline preoperative SpO_2_ data were collected.

Patients were premedicated (intravenous midazolam, 1–2 mg) and monitored using non-invasive arterial pressure, electrocardiogram, SpO_2_, and bispectral index (BIS, Covidien, Dublin, Ireland). General anaesthesia was performed using sevoflurane or propofol at discretion of attending anaesthesiologists, and cisatracurium was administered as a bolus (0.2 mg/kg) to allow orotracheal intubation and in continuous intravenous perfusion during surgery to ensure the lowest possible intraperitoneal pressure possible during laparoscopy. Intraoperative alveolar recruitment maneuvers (ARM) were performed at the anesthesiologist’s discretion, typically in response to a drop in oxygen saturation below 90%. During these maneuvers, the ventilator was switched to pressure-controlled ventilation with a driving pressure of 20 cm H_2_O and an initial positive end-expiratory pressure (PEEP) of 5 cm H_2_O. Subsequently, PEEP was increased in 5 cm H_2_O increments every 10 respiratory cycles, up to a maximum of 20 cm H_2_O. Patients were ventilated intraoperatively using volume control ventilation. At the end of surgery, neuromuscular reversal was administered. Neuromuscular reversal was performed at the end of surgery, before extubation.

Intraoperative ventilatory parameters were also recorded: tidal volume in ml per kg of predicted body weight, PEEP, peak pressure, compliance, inspired fraction of oxygen (FiO_2_), intraoperative ARMs, and endotracheal tube removal with or without positive pressure. The duration of surgery and intraoperative bleeding were also recorded.

Patients were transferred to the PACU, where an independent clinician was in-charge. In the absence of allergies, all patients received an intravenous continuous infusion of 12 g of metamizole and 300 mg of tramadol during the first 48 postoperative hours. Regional anesthesia techniques were not routinely employed. Thirty minutes after extubation, the visual analogue scale (VAS) was recorded based on the patient indicating a point on a 10 cm horizontal line representing his experience of pain. According to this scale, analgesic treatment was administered when postoperative pain was greater than mild (VAS 1–3) to achieve VAS lower than 3. Patients received supplemental oxygen through a venturi mask with FiO_2_ of 0.5 for the first 30 min. The Air-Test was then performed by removing the oxygen mask and leaving the patients breathing room air for at least 5 min while continuously monitoring SpO_2_ with a pulse oximeter finger probe [14]. The Air Test was then classified as positive (SpO_2_ ≤ 96%) or negative (SpO_2_ ≥ 97%). During the following 7 postoperative days, respiratory complications were recorded according to the definitions proposed by the Joint Task Force of the European Society of Anaesthesiology and the European Society of Intensive Care Medicine (ESCIM) [15]. Other complications were recorded: cardiovascular (arrhythmia, cardiogenic pulmonary oedema, myocardial infarction), infectious (infection, surgical site infection and urinary tract infection), and others (delirium, acute kidney injury, paralytic ileus [15]. Finally, hospital stay was collected. Postoperative complications and outcomes were collected by an investigator who was independent from the one performing the Air Test.

### 2.2. Statistical Analysis

Based on an expected incidence of postoperative pulmonary complications (PPCs) of 18% and an absolute precision of 5%, an initial sample size of 227 patients was calculated. Accounting for an anticipated 10% loss to follow-up, the adjusted sample size was determined to be 249 patients. Continuous variables were expressed as mean ± standard deviation and categorical variables as number (percent). Continuous variables in the PPC and non-PPC groups were compared using the student’s t-test or Mann–Whitney U test. Categorical variables in the PPC and non-PPC groups were compared using the chi-square test or Fisher’s exact test. Pearson’s correlation coefficient was used to assess the relationship between postoperative SpO_2_ and other quantitative variables. All analyses were exploratory without predefined gatekeeping procedures. Univariate and multivariate logistic regression analyses were performed to identify independent risk factors of PPCs. Variables with a *p*-value < 0.05 in univariate logistic regression analysis were entered into multivariate logistic regression analysis. A *p* < 0.05 was considered statistically significant. ROC and cross-tabs analyses were used to evaluate the sensitivity and specificity of Positive Air Test. Pearson’s correlation coefficient was used to correlate two quantitative variables. The data were analyzed using SPSS 24.0.

## 3. Results

**Figure 1:**
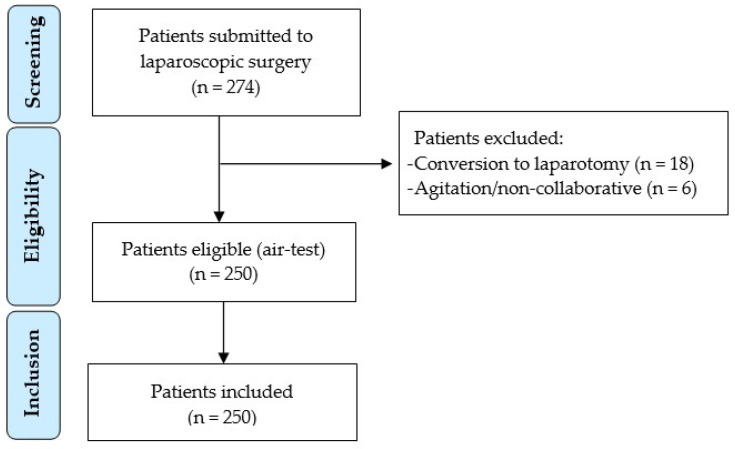
Patient flow chart diagram.

A total of 250 patients with a mean age of 61 ± 15 years were included. Twenty-four patients were excluded because they required conversion to laparotomy or had postoperative agitation/were uncooperative (Figure 1).

The characteristics of the 250 patients analysed and intraoperative data shown in Table 1 and Table 2.

Of these 250 patients, 36 (14.4%) had PPCs, and 214 (85.6%) did not. The most frequently observed PPCs were atelectasis, occurring in 32 patients (12.8%), followed by respiratory failure in 11 patients (4.4%), pleural effusion in 4 patients (1.6%), and aspiration pneumonitis in 1 patient (0.4%).

Univariate logistic regression analysis showed that age over 60 years and a positive Air Test were significantly associated with PPCs in patients undergoing laparoscopic surgery (Table 3). Multivariate logistic regression analysis confirmed these findings, with age >60 years (OR: 2.29; *p* = 0.041) and a positive Air Test (OR: 6.22; *p* = 0.001) remaining significantly associated with the occurrence of PPCs (Table 3). Preoperative SpO_2_ was lower in patients with a positive Air Test (97.3 ± 1.6 vs. 98.0 ± 1.4, *p* < 0.001). There was a positive correlation between preoperative and postoperative SpO_2_ (Air Test) (r =0.329, *p* < 0.001). A positive postoperative air test was significantly correlated with higher BMI (r = 0.137, *p* = 0.003), ARISCAT score (r = 0.175, *p* = 0.006), compliance (r = 0.251, *p* < 0.0001), and lower peak airway pressure (r = –0.206, *p* = 0.001). No correlation was found with the other variables.

**Figure 2:**
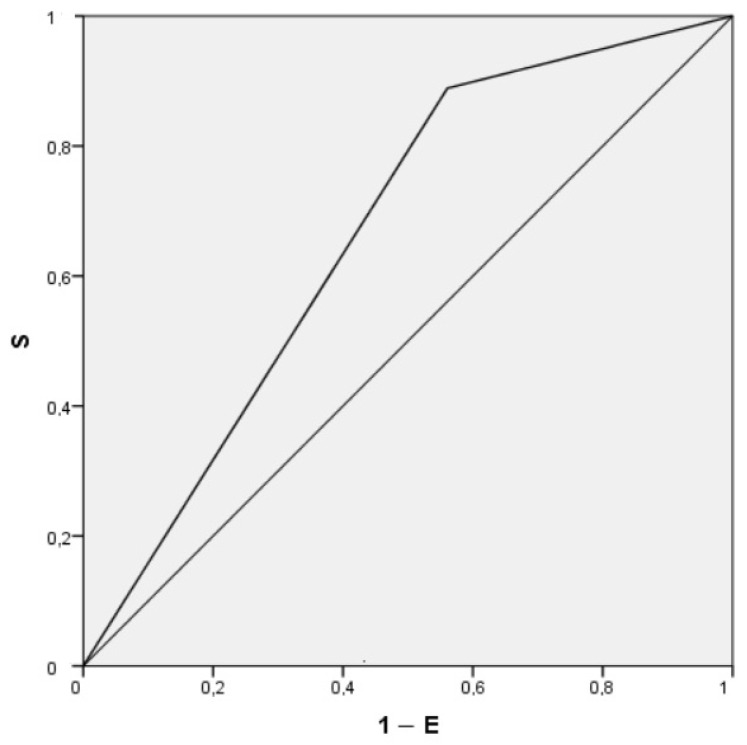
ROC (receiver operating characteristic) curve for Air Test predicting postoperative pulmonary complications. The area under the curve (AUC) of the air test was 0.664 (95% confidence interval: 0.580–0.746; *p* = 0.002) for predicting postoperative pulmonary complications (S: sensitivity; E: specificity).

The Air Test showed acceptable discriminative ability, with an area under the ROC curve (AUC) of 0.664 (95% CI: 0.580–0.746; *p* = 0.002), indicating statistically significant performance above chance (Figure 2). Using the optimal cutoff point, the sensitivity was 56%, specificity 11%, positive predictive value (PPV) 78.9%, and negative predictive value (NPV) 75%.

The mean VAS score reported 30 min after extubation was 3.46 ± 2.0. The Air Test was positive in 152 patients (60.8%), with a significantly higher proportion in the PPC group (88.9%), compared to the non-PPC group (56.1%). Infectious postoperative complications, postoperative nausea and vomiting, and adynamic ileus, as well as PACU stay and total hospital length of stay, were all significantly higher in the PPC group compared to the non-PPC group (Table 4). According to the type of surgery, the mean hospital stay was as follows: 2.5 ± 3.5 days for laparoscopic cholecystectomy, 6.5 ± 10 days for colorectal surgery, 3.3 ± 6.3 days for bariatric surgery, 5.4 ± 10 days for gastrectomy, and 4.3 ± 4.3 days for hepatic surgery.

## 4. Discussion

In this prospective study, the incidence of PPCs following laparoscopic surgery was 14.4%, with a higher prevalence observed in older patients and in those with a positive postoperative Air Test. Moreover, the development of PPCs was associated with a statistically significant increase in both PACU and overall hospital length of stay.

The incidence rate of PPCs in this study was 14.4%. In laparoscopic general surgery, available data on the incidence of PPCs are scarce, definitions of PPC vary considerably, and patient populations can differ markedly. Ntutumu et al. [16] reported a CPP incidence of 6.8% among gastric cancer patients undergoing laparoscopic gastrectomy, a predominantly male cohort (68.2%), which has been associated with a higher risk of CPP in some studies. Furthermore, their definition of PPC was not standardized and included only pneumonia, pleural effusion, and pulmonary embolism during the first 30 days, excluding atelectasis. This exclusion may partly explain why their reported PPC rate is lower than ours, since postoperative atelectasis is common in abdominal surgery and was not considered in their analysis. Gong et al. [17], in a recent study, reported a PPC incidence of approximately 14.9% among patients undergoing laparoscopic gastrectomy for gastric cancer. This rate closely matches our results. Their cohort, like that of Ntutumu et al. [16], consisted exclusively of patients undergoing laparoscopic surgery for gastric cancer, with a male predominance of around 68.3%. They used the same definition of PPC as our study, but for a period of 30 days. In robotic-assisted laparoscopic surgery, the incidence may increase to as high as 30% [18]. This incidence is relatively high. However, as the authors themselves clarify, it may have been influenced by the characteristics of the patient population, as all patients were older men, both of which are recognized risk factors for PPCs.

As predicted by the preoperative respiratory complication scores, our population consisted of patients at intermediate risk of complications. The mean ARISCAT score was 33 ± 12, corresponding to an intermediate risk category, with an estimated incidence of respiratory complications of approximately 13% [13]. The predicted complication rate is similar than that observed in our study (14.4%). We may reasonably expect a lower incidence of PPCs in our study population than predicted by the ARISCAT risk model, given that our cohort consisted exclusively of patients undergoing laparoscopic surgery, a factor not included in the ARISCAT score. The original ARISCAT study, published in 2010, included a heterogeneous population undergoing various types of surgical procedures under different anesthetic techniques, without accounting for the surgical approach (open vs. laparoscopic). At the time, laparoscopic techniques were not as prevalent as they are today. It is plausible that ARISCAT may overestimate PPC risk in cohorts limited to minimally invasive procedures.

However, the surgical site may be more relevant than the surgical technique in affecting diaphragmatic function and, consequently, the development of atelectasis and PPCs. Indeed, laparoscopic upper abdominal surgery has been shown to impair diaphragmatic excursion and is associated with higher rates of PPCs [19,20]. In our cohort, 37.2% involved laparoscopic upper abdominal procedures. Consistent with previous studies, we observed a higher incidence of PPCs in upper versus lower abdominal surgeries. Furthermore, our findings also support prior evidence showing that longer surgical duration is significantly associated with increased risk of PPCs [21]. This may be explained by the fact that longer surgical times are associated with increased formation of atelectasis and ventilation/perfusion mismatch.

In our study, we found that the mean tidal volume adjusted for predicted body weight was 8 mL/kg, with an average PEEP of 7 cm H_2_O. No significant differences were observed in ventilatory parameters between patients with and without PPCs, which may be attributed to the low variability in clinical practice regarding intraoperative ventilator settings. Additionally, we observed that approximately one-quarter of anesthesiologists reported using ARMs during surgery. The detrimental effects of mechanical ventilation in patients under general anaesthesia may lead to mechanical ventilation-associated lung injury and, therefore, an increase in PPCs [22]. A protective ventilation strategy in critically ill patients with adult respiratory distress syndrome has been recommended, including the use of low tidal volumes (4–8 mL/kg predicted body weight) and high levels of PEEP [22]. For prolonged ARMs, experts do not currently recommend their use [23,24]. For several years, researchers have been investigating whether this ventilatory strategy should be applied to the surgical patient. We have long known that optimal intraoperative ventilation strategies for surgical patients without severe lung injury must include low tidal volumes, but the use of moderate PEEP and ARMs has been more controversial [25]. A recent meta-analysis confirms that, in addition to the use of low tidal volumes, the use of PEEP ≥ 5 cmH_2_O also reduces atelectasis and PPCs [22]. However, it remains to be confirmed that ARMs reduce PPCs.

In laparoscopic surgery, there is also a randomized controlled trial in adult patients demonstrating that intraoperative mechanical ventilation with low tidal volumes (6 mL/kg predicted body weight) was associated with a significant reduction in PPC compared with conventional tidal volume ventilation [26]. Mechanical power is a new concept that includes several ventilatory parameters such as peak pressure, plateau pressure, respiratory rate, PEEP and tidal volume. Like other predictors of mechanical ventilation, it has emerged in ICU patients with ARDS and is associated with high morbidity and mortality in ICU patients. In the surgical population, particularly in patients undergoing general anaesthesia for elective surgery, high intraoperative mechanical power ventilation has recently been demonstrated to be a risk factor for the development of postoperative pulmonary complications [27].

Age greater than 60 years was identified as an independent predictor of PPCs in our study. This finding is consistent with previous research [18], which has shown that advancing age is associated with an increased risk of PPCs, and, indeed, age is also one of the items included in the ARISCAT and LAS VEGAS risk scores.

Additionally, a positive Air Test was also found to be an independent predictor of PPCs in the postoperative period. The Air Test is a simple and inexpensive test that can be performed at the bedside and has been shown to detect postoperative atelectasis. Atelectasis affects up to 90% of surgical patients [28]. Administration of oxygen to all patients in the immediate postoperative period as routine clinical management may allow SpO_2_ reductions to go unnoticed. Positive Air Test (SpO_2_ is ≤ 96%) has a sensitivity of 82.6% and specificity of 87.8% for the diagnosis of atelectasis [29]. One of the most important factors in the development of PPCs is the presence of atelectasis in the immediate postoperative period [30]. Therefore, identifying patients with early postoperative atelectasis may help predict which individuals are at higher risk of developing PPCs. A positive Air Test can help clinicians initiate early measures to treat atelectasis, such as optimizing analgesic management; incentivizing spirometry, deep breathing exercises, or secretion mobilization; and the application of continuous positive airway pressure and inspiratory muscle training. All of these measures can lead to better patient outcomes, shorter hospital stays, and a reduced likelihood of long-term complications [31].

A recent study also found that the Air Test was an independent predictor of PPCs following major abdominal surgery [9]. However, unlike our study, it also included laparotomies in addition to laparoscopic approach. Given that the incidence of PPCs is lower in laparoscopic surgery compared to laparotomies, it seems relevant to study laparoscopic surgery separately. In this context of laparoscopic surgery, where the baseline risk of PPCs is relatively low, the identification of a sensitive and specific predictive tool becomes particularly relevant. To our knowledge, this is the first study to identify the Air Test as an independent predictor of PPCs in the setting of laparoscopic surgery.

Our incidence of a positive Air Test was 60%, higher than that reported by Ferrando et al. (36.4%) [14]. The lower incidence found may have occurred, because the authors excluded patients with a preoperative SpO_2_ ≤ 97% breathing room air. As we observed in our study, preoperative SpO_2_ correlates with postoperative SpO_2_. Therefore, by excluding these patients, the incidence of positive Air Test should be lower. In our opinion, it is more interesting to include patients with low preoperative SpO_2_, as they have been shown to have a higher incidence of PPCs [32]. Indeed, both LAS VEGAS [33] and ARISCAT [13,34] include preoperative SpO_2_ as independent risk factors for PPCs. Consistent with these studies, we also found that patients with lower preoperative SpO_2_ had more positive test results.

A follow-up study by the original developers of the Air Test demonstrated that combining pre- and postoperative room air SpO_2_ values provides a reasonably accurate prediction of PPCs [29]. In our study, preoperative SpO_2_ was not an independent predictor of PPCs. They observed more atelectasis than we did (25.9% vs. 12.8%) but a lower percentage of positive Air Test (34 vs. 60%). In this study, patients underwent a CT scan 25 min after the test to detect atelectasis. In this observational study, atelectasis was confirmed only in cases where imaging was performed for other clinical indications, such as chest X-rays for central line verification or CT scans prompted by suspected surgical complications. Furthermore, the population included in this study was different from ours, as one of the inclusion criteria was an intermediate-to-high risk of PPCs, according to the ARISCAT scale. In contrast to our study, patients who underwent laparotomy were also included.

Given the potential advantages of the Air Test and the results of our multivariate analysis, we investigated the discriminative ability of the Air Test to predict whether a patient will develop PPCs following laparoscopic surgery. Although the Air Test demonstrated statistically significant performance above chance, with an AUC of 0.664 (95% CI: 0.580–0.746; *p* = 0.002), its overall discriminative ability was limited. The optimal cutoff yielded modest sensitivity (56%) and very low specificity (11%), indicating a high rate of false positives. The Air Test showed a high positive predictive (PPV 78.9), despite low specificity, likely due to the relatively high prevalence of the outcome in our population. Although sensitivity was reasonable, some false positives occurred. This can be explained by the fact that a positive Air Test may reflect the presence of postoperative atelectasis, which, if persistent, could predispose patients to other types of PPCs. However, in most cases, postoperative atelectasis resolves within the first few hours, often before there is sufficient time for secondary PPCs to develop. These findings suggest that, although the Air Test may be useful as a preliminary screening tool, it lacks sufficient accuracy for standalone clinical decision-making. Ferrando et al. [29] reported that the Air Test had a better discriminative ability, with an AUC of 0.72 (95% CI: 0.67–0.76), particularly when combined with preoperative SpO_2_. In our study, however, preoperative SpO_2_ did not differ between groups, and we were unable to identify a clear explanation for this finding.

In our study, the PPC group presented a higher incidence of infectious and minor complications, such as adynamic ileus and postoperative nausea and vomiting. This group also had a longer stay in the PACU and hospital. Although these findings have been previously described in the literature [35], they have not been specifically reported in the setting of laparoscopic surgery.

We acknowledge several strengths of this study, including the fact that it was conducted exclusively on laparoscopic surgery. Additionally, PPCs were defined according to the criteria proposed by the Joint Task Force of the European Society of Anaesthesiology and the European Society of Intensive Care Medicine (ESICM), allowing for comparability with other studies. Regarding the limitations of this study, it would have been valuable to record additional intraoperative parameters, such as plateau pressure, driving pressure, and pneumoperitoneum pressure in order to explore their potential association with PPCs. Another limitation is that, although neuromuscular blockade reversal is routinely performed in our center, on some occasions, we are unable to monitor it, so we cannot guarantee 100% reversal. Furthermore, the use of lung ultrasound for the diagnosis of atelectasis would have been of interest, given its high sensitivity and non-invasive nature. In the near future, the standardization of bedside lung ultrasound upon ICU or PACU admission in the immediate postoperative period may improve the early detection of atelectasis and enhance the prediction of PPCs.

## 5. Conclusions

Laparoscopy has become a routine approach to the abdominal cavity in the twenty-first century. Even in laparoscopic surgery, a minimally invasive technique, there is a high risk of atelectasis and PPCs. This prospective observational study found that up to 14% of patients undergoing laparoscopic surgery developed PPCs, with a higher incidence observed in upper abdominal procedures and surgeries of longer duration. In addition, patients who developed PPCs had longer stays in both the post-anesthesia care unit (PACU) and the hospital. As a novel finding, our study identified a positive postoperative Air Test as an independent risk factor for PPCs following laparoscopic surgery. Although the Air Test remains a practical and non-invasive bedside tool for the early detection of atelectasis, its diagnostic accuracy in this setting was limited, with modest discriminative capacity. These results highlight the need for further investigation to validate the clinical utility of the Air Test specifically in patients undergoing laparoscopic procedures.

## Figures and Tables

**Table 1:** Characteristics of patients.

Study Population		All Patients (n = 250)	PPC Group (n = 36)	Non PPC Group (n = 214)	*p*-Value
Age, years		61.5 ± 14.6	68.6 ± 8.8	60.4 ± 15	0.02
Male, n (%)		137 (54.8)	117 (54.7)	20 (55.6)	0.922
BMI, kg·m^−1^		27.8 ± 5.8	27.1 ± 4.6	28 ± 5.9	0.373
ASA physical status	1, n (%)	19 (7.6)	0	19(8.9)	0.185
	2, n (%)	141 (56.4)	20 (55.6)	121 (56.5)	
	3, n (%)	87 (34.8)	16 (44.4)	71 (32.3)	
	4, n (%)	3 (1.2)	0 (0)	3 (1.4)	
ARISCAT score		33.2 ± 12.6	33.5 ± 12.5	33.5 ± 12.7	0.914
FRAIL Score		1.52 ± 0.64	1.58 ± 0.6	1.51 ± 0.63	0.549
Preoperative SpO_2_, %		97.6 ± 1.5	97.3 ± 1.67	97.6 ± 1.5	0.209
Smoking status	Smoker, n (%)	62 (24.8)	10 (27.8)	52 (24.3)	0.678
	Nonsmoker, n (%)	188 (75.2)	26 (72.2)	162 (75.7)	
Comorbidities	COPD, n (%)	12 (4.8)	2 (5.6)	10 (4.7)	0.685
	Arterial hypertension, n (%)	124 (49.6)	19 (52.8)	105 (49.1)	0.721
	Diabetes Mellitus, n (%)	66 (26.4)	11 (30.6)	55 (25.7)	0.544
	Ischemic heart disease, n (%)	8 (3.2)	0 (0)	8(3.7)	0.607
	Obesity, n (%)	60 (24)	6 (16.7)	54 (25.2)	0.3
	Dyslipidaemia, n (%)	119 (47.6)	21 (58.3)	98 (45.8)	0.207
Indication for surgery	Colorectal cancer, n (%)	93 (37.2)	11(30.5)	82 (38.3)	0.563
	Cholelithiasis, n (%)	83 (33.2)	13 (36.1)	70 (32.7)	
	Bariatric surgery, n (%)	25 (10)	4 (11.1)	21(8.7)	
	Gastric cancer, n (%)	45 (18)	7 (19.4)	38 (17.7)	
	Liver cancer, n (%)	4 (1.6)	2 (0.5)	2 (0.93)	

Data are expressed as mean ± SD, or frequency (%). PPC: postoperative pulmonary complications; BMI: body mass index; ASA: American Society of Anesthesiologists; ARISCAT: assess respiratory risk in surgical patients in Catalonia; SpO_2_: oxygen saturation as measured by pulse oximetry; COPD: chronic obstructive pulmonary disease.

**Table 2:** Intraoperative data and mechanical ventilation parameters.

	All Patients (n = 250)	PPC Group (n = 36)	Non PPC Group (n = 214)	*p*-Value
Upper abdominal surgery, n (%)	93 (37.2)	18(50)	75 (35)	0.086
Intraoperative bleeding, mL	165.5 ± 156.5	202.5 ± 169	159.3 ± 153	0.126
Length of surgery, min	142.7 ± 108.1	173.4 ± 133	135 ± 86.3	0.025
Tidal volume predicted body weight, mL·kg^−1^	7.8 ± 1.2	7.9 ± 1.1	7.7 ± 1.2	0.570
PEEP, cmH_2_O	6.5 ± 1.9	6.69 ± 2.2	6.49 ± 1.84	0.555
Peak pressure, cmH_2_O	23 ± 4.5	22.81 ± 5.5	23.20 ± 4.4	0.638
FiO_2_, %	57.4 ± 9.3	57.42 ± 7.5	57.42 ± 9.6	0.998
Compliance, mL·cmH_2_O^−1^	34.7 ± 8.9	33.34 ± 8.5	34.95 ± 9	0.319
Alveolar recruitment maneuver, n (%)	64 (25.6)	11 (30.6)	53 (24.8)	0.536
Withdrawal ETT with positive pressure, n (%)	98 (39.2)	12 (33.3)	89 (40.2)	0.467

Data are expressed as mean ± SD, or frequency (%). PPC: postoperative pulmonary complications; PEEP: positive end-expiratory pressure; FiO_2_: fraction of inspired oxygen; ETT: endotracheal tube.

**Table 3:** Univariate and multivariate logistic regression analyses of factors predictive of postoperative pulmonary complications.

Variables	Univariate Analysis		Multivariate Analysis	
	OR (95%CI)	*p*-Value	OR (95%CI)	*p*-Value
Preoperative variables				
Male	0.965 (0.474–1.96)	0.922		
Age > 60 years	2.34 (1.07–5.10)	0.032	2.29 (1.03–5.08)	0.041
BMI >30, kg·m^−2^	2.34 (1.07–5.10)	0.423		
COPD	0.83 (0.17–3.9)	0.8119		
Smoker	0.83 (0.37–1.85)	0.655		
ARISCAT score	0.82 (0.46–1.46)	0.519		
Preoperative SpO_2_ < 96%	1.72 (0.32–1.22)	0.172		
Arterial hypertension	1.22 (0.61–1.82)	0.765		
Diabetes mellitus	0.83 (0.56–1.95)	0.983		
Ischemic heart disease	2.14 (1.12–4.10)	0.125		
Dyslipidaemia	0.53 (0.41–1.95)	0.111		
Obesity	2.11 (1.71–3.30)	0.238		
Intraoperative variables				
Lower laparoscopic surgery	1.85 (0.1–3.77)	0.089		
Alveolar recruitment maneuver	0.74 (0.34–1.62)	0.463		
Withdrawal ETT with positive pressure	1.34 (0.63–2.89)	0.437		
Tidal volume predicted body weight, mL·kg^−1^	1.15 (0.53–2.47)	0.567		
PEEP, cmH_2_O	1.24 (0.73–2.69)	0.456		
FiO_2_, %	1.05 (0.23–1.47)	0.234		
Peak pressure, cmH_2_O	2.15 (0.53–2.47)	0.952		
Compliance < 30, mL·cmH_2_O^−1^	1.15 (0.53–2.47)	0.831		
Postoperative variables				
Positive Air Test	6.26 (2.14–18.34)	0.001	6.22 (2.11–18.22)	0.001

Data are expressed as mean ± SD, or frequency (%). OR: odds ratio; CI: confidence interval; BMI: body mass index; ARISCAT: assess respiratory risk in surgical patients in Catalonia; SpO_2_: oxygen saturation as measured by pulse oximetry; COPD: chronic obstructive pulmonary disease; FiO_2_: fraction of inspired oxygen; ETT: endotracheal tube; PEEP: positive end-expiratory pressure.

**Table 4:** Postoperative data.

	All Patients (n = 250)	PPC Group (n = 36)	Non PPC Group (n = 214)	*p*-Value
VAS	3.46 ± 2	3.89 ± 2.2	3.39 ± 2	0.185
Air test, %	95.4 ± 2.98	93.97 ± 3	95.68 ± 2.9	0.001
Positive Air test, n (%)	152 (60.8)	32 (88.9)	120 (56.1)	0.000
Cardiovascular complications, n (%)	12 (4.8)	2 (5.6)	10 (4.7)	0.685
Infectious complications, n (%)	37 (14.8)	11 (30)	26 (12.1)	0.009
Other complications, n (%)				
Adynamic ileus	13 (5.2)	5 (13.8)	8 (3.7)	0.025
Postoperative nausea and vomiting	44 (17.6)	10 (27.7)	34 (15.8)	0.098
Delirium	4 (1.6)	2 (5.8)	2 (0.9)	0.1
Acute kidney injury	7 (2.8)	2 (5.8)	5 (2.3)	0.266
PACU stay, hours	9 ± 15.4	15.9 ± 25.8	7.95 ± 12.7	0.005
Hospital stay, days	4.63 ± 5.7	8.2 ± 11.8	4 ± 3.6	0.000

Data are expressed as mean ± SD, or frequency (%). PPC: postoperative pulmonary complications; VAS: visual analog scale, PACU: post-anesthesia care unit.

## Data Availability

The original contributions presented in this study are included in the article. Further inquiries can be directed to the corresponding author.

## Abbreviations

The following abbreviations are used in this manuscript:

PPCs	Postoperative pulmonary complications
VAS	Visual analogue scale
PACU	Post-anesthesia care unit

## References

[1] Gerges F.J., Kanazi G.E., Jabbour-Khoury S.I. (2006). Anesthesia for laparoscopy: A review. J. Clin. Anesth..

[2] Riemma G., De Franciscis P., La Verde M., Ravo M., Fumiento P., Fasulo D.D., Della Corte L., Ronsini C., Torella M., Cobellis L. (2023). Impact of the hemostatic approach after laparoscopic endometrioma excision on ovarian reserve: Systematic review and network meta-analysis of randomized controlled trials. Int. J. Gynaecol. Obstet..

[3] Dokmak S., Scatton O., Fusco G., Belghiti J., Gayet B., Soubrane O. (2016). Laparoscopy Decreases Pulmonary Complications in Patients 4Undergoing Major Liver Resection: A Propensity Score Analysis. Ann. Surg..

[4] Antoniou S.A., Antoniou G.A., Koch O.O., Köhler G., Pointner R., Granderath F.A. (2015). Laparoscopic versus open obesity surgery: A meta-analysis of pulmonary complications. Dig. Surg..

[5] Guo Y., Cao F., Ding Y., Sun H., Liu S., Li A., Li F. (2019). Laparoscopic Major Gastrointestinal Surgery Is Safe for Properly Selected Patient with COPD: A Meta-Analysis. Biomed. Res. Int..

[6] Hasukić Š., Mešićć D., Dizdarević E., Keser D., Hadžiselimović S., Bazardžanović M. (2002). Pulmonary function after laparoscopic and open cholecystectomy. Surg. Endosc..

[7] Bablekos G.D., Michaelides S.A., Analitis A., Charalabopoulos K.A. (2014). Effects of laparoscopic cholecystectomy on lung function: A systematic review. World J. Gastroenterol..

[8] Ferrando C., Soro M., Unzueta C., Suarez-Sipmann F., Canet J., Librero J., Pozo N., Peiró S., Llombart A., León I. (2018). Individualized PeRioperative Open-lung VEntilation (iPROVE) Network. Individualised perioperative open-lung approach versus standard protective ventilation in abdominal surgery (iPROVE): A randomised controlled trial. Lancet Respir. Med..

[9] Ferrando-Ortolá C., iPROVE Research Network Group for the PEALS study (2025). Postoperative pulmonary complications in emergency abdominal surgery. A prospective international cohort study. Anaesth. Crit. Care Pain Med..

[10] Chandler D., Mosieri C., Kallurkar A., Pham A.D., Okada L.K., Kaye R.J., Cornett E.M., Fox C.J., Urman R.D., Kaye A.D. (2020). Perioperative strategies for the reduction of postoperative pulmonary complications. Best. Pract. Res. Clin. Anaesthesiol..

[11] Miskovic A., Lumb A.B. (2017). Postoperative pulmonary complications. Br. J. Anaesth..

[12] Fernandez-Bustamante A., Frendl G., Sprung J., Kor D.J., Subramaniam B., Martinez-Ruiz R., Lee J.-W., Henderson W.G., Moss A., Mehdiratta N. (2017). Postoperative Pulmonary Complications, Early Mortality, and Hospital Stay Following Noncardiothoracic Surgery: A Multicenter Study by the Perioperative Research Network Investigators. JAMA Surg..

[13] Canet J., Gallart L., Gomar C., Paluzie G., Vallès J., Castillo J., Sabate S., Mazo V., Briones Z., Sanchis J. (2010). Prediction of postoperative pulmonary complications in a population-based surgical cohort. Anesthesiology.

[14] Ferrando C., Romero C., Tusman G., Suarez-Sipmann F., Canet J., Dosdá R., Valls P., Villena A., Serralta F., Jurado A. (2017). The accuracy of postoperative, noninvasive Air-Test to diagnose atelectasis in healthy patients after surgery: A prospective, diagnostic pilot study. BMJ Open.

[15] Jammer I.B., Wickboldt N., Sander M., Smith A., Schultz M.J., Pelosi P., Leva B., Rhodes A., Hoeft A., Walder B. (2015). European Society of Anaesthesiology (ESA) and the European Society of Intensive Care Medicine (ESICM); Standards for definitions and use of outcome measures for clinical effectiveness research in perioperative medicine: European Perioperative Clinical Outcome (EPCO) definitions: A statement from the ESA-ESICM joint taskforce on perioperative outcome measures. Eur. J. Anaesthesiol..

[16] Ntutumu R., Liu H., Zhen L., Hu Y.F., Mou T.Y., Lin T., Yu J., Li G.X. (2016). Risk factors for pulmonary complications following laparoscopic gastrectomy: A single-center study. Medicine.

[17] Gong J., Xu L., Yu H., Qiu F., Zhang Z., Yin Y., Ma H., Cai Z., Zhong J., Ding W. (2024). Increased postoperative complications after laparoscopic gastrectomy in patients with preserved ratio impaired spirometry. J. Gastrointest. Surg..

[18] Yu J., Park J.Y., Kim D.H., Kim S., Hwang J.H., Seo H., Kim Y.-K. (2019). Incidence and Risk Factors of Pulmonary Complications after Robot-Assisted Laparoscopic Prostatectomy: A Retrospective Observational Analysis of 2208 Patients at a Large Single Center. J. Clin. Med..

[19] Erice F., Fox G.S., Salib Y.M., Romano E., Meakins J.L., Magder S.A. (1993). Diaphragmatic function before and after laparoscopic cholecystectomy. Anesthesiology.

[20] Ferreyra G., Long Y., Ranier V.M. (2009). Respiratory complications after major surgery. Curr. Opin. Crit. Care.

[21] Brooks-Brunn J.A. (1997). Predictors of postoperative pulmonary complications following abdominal surgery. Chest.

[22] Deng Q.W., Tan W.C., Zhao B.C., Wen S.H., Shen J.T., Xu M. (2020). Intraoperative ventilation strategies to prevent postoperative pulmonary complications: A network meta-analysis of randomised controlled trials. Br. J. Anaesth..

[23] Grasselli G., Calfee C.S., Camporota L., Poole D., Amato M.B.P., Antonelli M., Arabi Y.M., Baroncelli F., Beitler J.R., Bellani G. (2023). ESICM guidelines on acute respiratory distress syndrome: Definition, phenotyping and respiratory support strategies. Intensive Care Med..

[24] Qadir N., Sahetya S., Munshi L., Summers C., Abrams D., Beitler J., Bellani G., Brower R.G., Burry L., Chen J.-T. (2023). An Update on Management of Adult Patients with Acute Respiratory Distress Syndrome: An Official American Thoracic Society Clinical Practice Guideline. Am. J. Respir. Crit. Care Med..

[25] Barbosa F.T., Castro A.A., de Sousa-Rodrigues C.F. (2014). Positive end-expiratory pressure (PEEP) during anesthesia for prevention of mortality and postoperative pulmonary complications. Cochrane Database Syst Rev..

[26] Karalapillai D., Weinberg L., Neto A.S., Peyton P.J., Ellard L., Hu R., Pearce B., Tan C.O., Story D., O’donnell M. (2023). Low tidal volume ventilation for patients undergoing laparoscopic surgery: A secondary analysis of a randomised clinical trial. BMC Anesthesiol..

[27] El-Khatib M., Zeeni C., Shebbo F.M., Karam C., Safi B., Toukhtarian A., Nafeh N.A., Mkhayel S., Shadid C.A., Chalhoub S. (2024). Intraoperative mechanical power and postoperative pulmonary complications in low-risk surgical patients: A prospective observational cohort study. BMC Anesthesiol..

[28] Wood C.B., Shinn J.R., Rees A.B., Patel P.N., Freundlich R.E., Smith D.K., McEvoy M.D., Rohde S.L. (2019). Existing Predictive Models for Postoperative Pulmonary Complications Perform Poorly in a Head and Neck Surgery Population. J. Med. Syst..

[29] Ferrando C., Suárez-Sipmann F., Librero J., Pozo N., Soro M., Unzueta C., Brunelli A., Peiró S., Llombart A., Balust J. (2020). A noninvasive postoperative clinical score to identify patients at risk for postoperative pulmonary complications: The Air-Test Score. Minerva Anestesiol..

[30] Ko E., Yoo K.Y., Lim C.H., Jun S., Lee K., Kim Y.H. (2023). Is atelectasis related to the development of postoperative pneumonia? a retrospective single center study. BMC Anesthesiol..

[31] Dhillon G., Buddhavarapu V.S., Grewal H., Munjal R., Verma R.K., Surani S., Kashyap R. (2023). Evidence-based Practice Interventions for Reducing Postoperative Pulmonary Complications: A Narrative Review. Open Respir. Med. J..

[32] Atilla N., Arpag H., Bozkus F., Kahraman H., Cengiz E., Bulbuloglu E., Atilla S. (2017). Can We Predict the Perioperative Pulmonary Complications Before Laparoscopic Sleeve Gastrectomy: Original Research. Obes. Surg..

[33] Neto A.S., da Costa L.G.V., Hemmes S.N.T., Canet J., Hedenstierna G., Jaber S., Hiesmayr M., Hollmann M., Mills G., Vidal Melo M. (2018). The LAS VEGAS risk score for prediction of postoperative pulmonary complications: An observational study. Eur. J. Anaesthesiol..

[34] Canet J., Sabaté S., Mazo V., Gallart L., de Abreu M.G., Belda J., Langeron O., Hoeft A., Pelosi P. (2015). Development and validation of a score to predict postoperative respiratory failure in a multicentre European cohort: A prospective, observational study. Eur. J. Anaesthesiol..

[35] Piccioni F., Spagnesi L., Pelosi P., Bignami E., Guarnieri M., Fumagalli L., Polati E., Schweiger V., Comi D., D’Andrea R. (2023). Postoperative pulmonary complications and mortality after major abdominal surgery. An observational multicenter prospective study. Minerva Anestesiol..

